# Immune response to SARS-CoV-2 in severe disease and long COVID-19

**DOI:** 10.1016/j.isci.2022.104723

**Published:** 2022-07-04

**Authors:** Tomonari Sumi, Kouji Harada

**Affiliations:** 1 Research Institute for Interdisciplinary Science, Okayama University, 3-1-1 Tsushima-Naka, Kita-ku, Okayama 700-8530, Japan; 2 Department of Chemistry, Faculty of Science, Okayama University, 3-1-1 Tsushima-Naka, Kita-ku, Okayama 700-8530, Japan; 3 Department of Computer Science and Engineering, Toyohashi University of Technology, Tempaku-cho, Toyohashi 441-8580, Japan; 4 Center for IT-Based Education, Toyohashi University of Technology, Tempaku-cho, Toyohashi, Aichi 441-8580, Japan

**Keywords:** immunology, virology, mathematical biosciences

## Abstract

COVID-19 is mild to moderate in otherwise healthy individuals but may nonetheless cause life-threatening disease and/or a wide range of persistent symptoms. The general determinant of disease severity is age mainly because the immune response declines in aging patients. Here, we developed a mathematical model of the immune response to SARS-CoV-2 and revealed that typical age-related risk factors such as only a several 10% decrease in innate immune cell activity and inhibition of type-I interferon signaling by autoantibodies drastically increased the viral load. It was reported that the numbers of certain dendritic cell subsets remained less than half those in healthy donors even seven months after infection. Hence, the inflammatory response was ongoing. Our model predicted the persistent DC reduction and showed that certain patients with severe and even mild symptoms could not effectively eliminate the virus and could potentially develop long COVID.

## Highlights

• Systemic SARS-CoV-2 infection owing to ACE2 expression on a wide range of cell types
• Persistent viral infection can be ongoing within the host even if it is not severe
• Long-term dendritic cell deficiency is owing to viruses that cannot be removed by the host
• Ongoing persistent viral infection within the host potentially causes long COVID or PASC

## Graphical abstract

**Figure:**
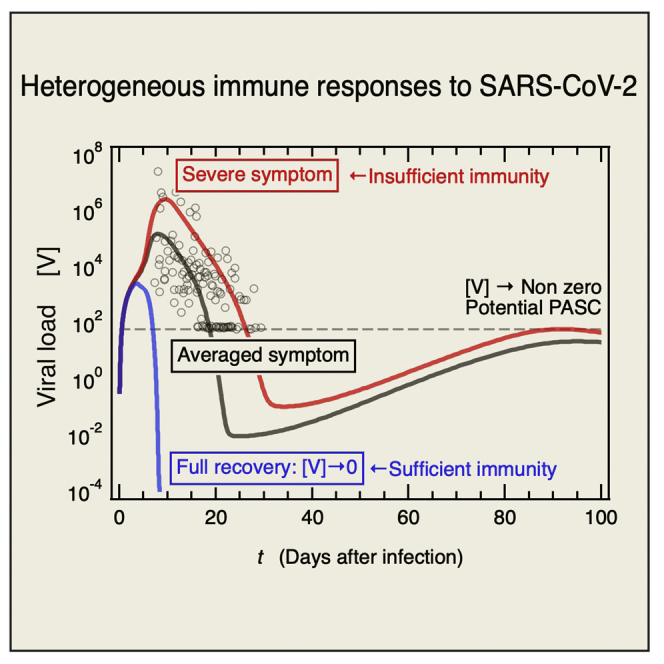

## Introduction

The emergence of severe acute respiratory syndrome coronavirus 2 (SARS-CoV-2) has caused an unprecedented ongoing global pandemic known as coronavirus 2019 (COVID-19) (J. F.-W. Chan et al., 2020; Zhou et al., 2020). The disease has heterogeneous characteristics. It may be asymptomatic, induce mild symptoms, or cause critical illness. In the latter case, 10–20% of all symptomatic patients are at elevated risks of multiple organ system involvement and mortality (Gupta et al., 2020; Huang et al., 2020; Zhou et al., 2020). There is limited experimental or clinical evidence that the virus *per se* is mainly responsible for the heterogeneity of the disease it causes. In contrast, there is growing evidence that the host accounts for the observed variability in disease severity, infection rate, and long-term disease symptoms (Schultze and Aschenbrenner, 2021). Therefore, a better understanding of the innate and adaptive immune response in mild and potentially fatal COVID-19 is crucial for developing diagnostic markers and therapeutic strategies.

**Figure 1:**
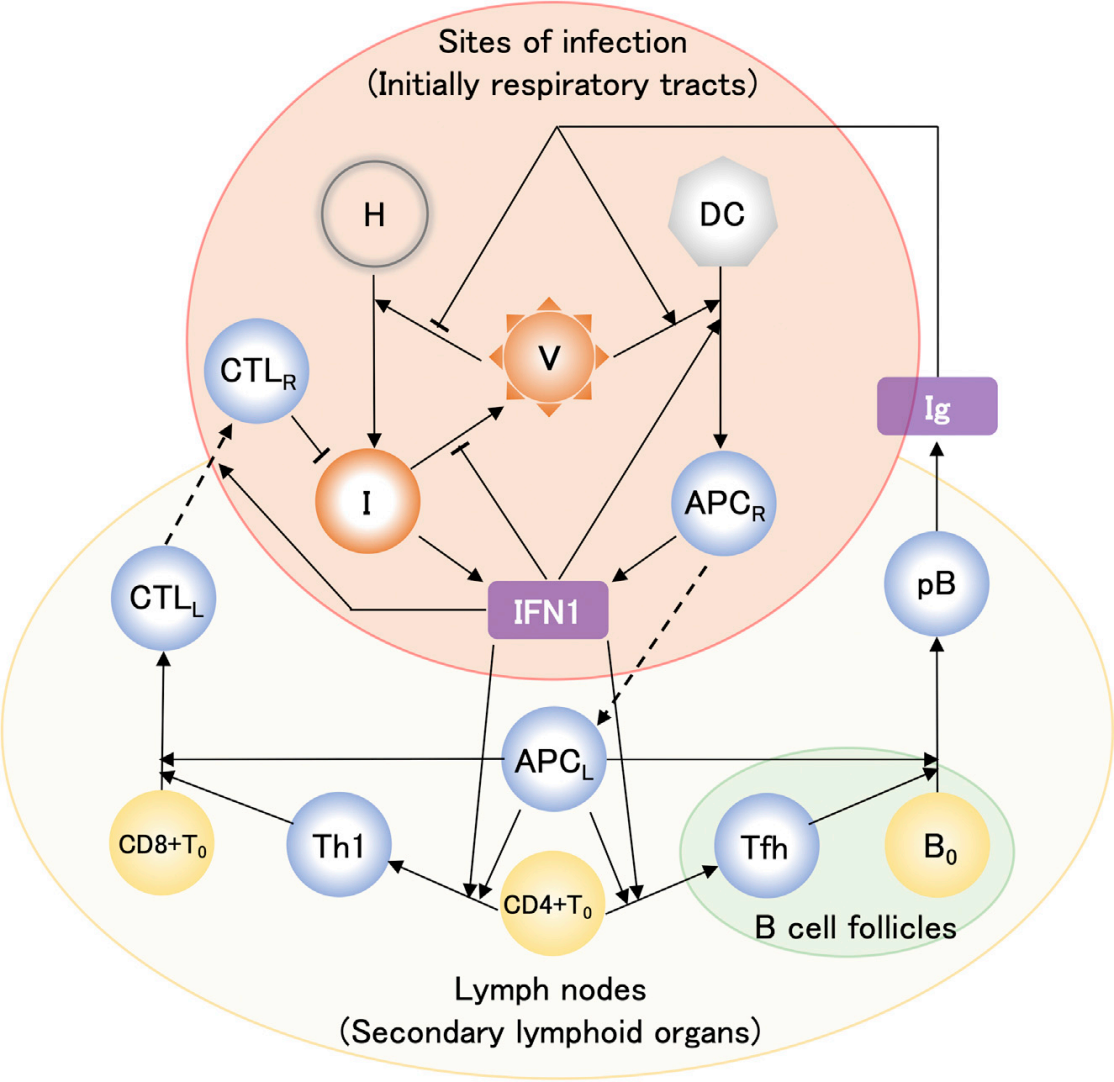
Mathematical model of host immune response to SARS-CoV-2 infection Solid arrow denotes either activation or differentiation. Dashed and blunt arrows denote migration and inhibition, respectively. Model variables include target healthy epithelial cells [H], infected cells [I], viral loads [V], dendritic cells [DC], antigen-presenting cells generated from DC at infection sites [APC_R_] and in lymph nodes [APC_L_], naive CD4^+^ and CD8^+^ T cells, [CD4^+^T_0_] and [CD8^+^T_0_], naive B cells [B_0_], type-I helper T cells [Th1], T follicular helper cells [Tfh], cytotoxic T lymphocytes in lymph nodes [CTL_L_] and infection sites [CTL_R_], plasma B cells [pB], type-I interferon [IFN1], and immunoglobulin [Ig]. The typical flow in the immune response depicted in this figure is as follows: The healthy epithelial cells are infected by viral particles and become infected cells. The infected cells produce viral particles, also secreting IFN1 molecules (Sa Ribero et al., 2020). DC cells ingest viral particles and become working as APC_R_ cells. The APC_R_ cells secrete IFN1 molecules (Fitzgerald-Bocarsly and Feng, 2007). The APC_R_ cells migrate toward lymph nodes. The moved APC_R_ cells, namely, APC_L_ cells differentiate CD4^+^T_0_ cells into Th1 and Tfh cells (Sette and Crotty, 2021), where IFN1 stimulates these developments (Cucak et al., 2009; Huber and Farrar, 2011). The APC_L_ and Th1 cells activate CD8^+^T_0_ cells, which then differentiate into CTL_L_ cells (Swain et al., 2012). The CTL_L_ cells are recruited by IFN1 to migrate toward the sites of infection and the moved CTL_L_ cells, namely, CTL_R_ cells kill infected cells (Sette and Crotty, 2021). The APC_L_ and Tfh cells activate B_0_ cells (Akkaya et al., 2020; Swain et al., 2012), which differentiate into pB cells, consequently Ig molecules are produced by the pB cells (Akkaya et al., 2020).

Many quantitative methods have been developed to analyze the dynamics of SARS-CoV-2 infection within the host (Almocera et al., 2021; Challenger et al., 2022; Chowdhury et al., 2022; Du and Yuan, 2020; Ejima et al., 2021; Ghosh, 2021; Hernandez-Vargas and Velasco-Hernandez, 2020; Kim et al., 2021; Moses et al., 2021; Nath et al., 2021; Perelson and Ke, 2021; Reis et al., 2021; Sahoo et al., 2020; Voutouri et al., 2021) and some also explicitly considered immune cells (Almocera et al., 2021; Challenger et al., 2022; Chowdhury et al., 2022; Du and Yuan, 2020; Ghosh, 2021; Moses et al., 2021; Reis et al., 2021; Sahoo et al., 2020; Voutouri et al., 2021). The mathematical modeling studies which theoretically show the instability of disease-free equilibrium and stability of the virus co-existence equilibrium (Almocera et al., 2021; Chowdhury et al., 2022; Ghosh, 2021; Nath et al., 2021) are suggestive of understanding clinically observed long-term symptoms of COVID-19. The development of large-scale models for simulating spatial-temporal dynamics of viral spread and immune response inside lungs (Moses et al., 2021) or incorporating the viral invasion process into epithelial cells, viral dissemination via the bloodstream, and systemic infection and thrombosis (Voutouri et al., 2021) is remarkable. The present study proposes a mathematical model for the immune response to SARS-CoV-2 incorporating the immune cells, related molecules, and their interactions (Figure 1). It reveals the roles of innate and adaptive immunity and examines the mechanisms of the development of severe COVID-19 in response to age-related risk factors. SARS-CoV-2 has already mutated to evade the immune response. For example, it dysregulates type-I interferon (IFN1) which is a cytokine secreted by infected host cells (Sa Ribero et al., 2020). Here, model simulations were used to assess age-related risk factors (Bastard et al., 2021), e.g., virally mediated suppression effect of IFN1 production by infected epithelial cells and the influences of IFN1-neutralizing autoantibodies.

Dendritic cells (DCs) are key orchestrators of the immune response. However, their functionality declines with age (Agrawal et al., 2007; Agrawal and Gupta, 2011; Sridharan et al., 2011). In contrast, aging does not substantially influence DC abundance or phenotype. Nevertheless, the capacity of DCs to phagocytose antigens and migrate is impaired with age (Agrawal et al., 2007; Agrawal and Gupta, 2011). In addition, the ability of DCs to present antigens to CD4^+^ and CD8^+^ T cells also decreases during the aging process (Sridharan et al., 2011). The model simulations also demonstrated the impact of these age-associated DC impairments on COVID-19 severity.

Influenza A virus infection generally causes self-limited bronchitis and possibly severe pneumonia (Miyazawa, 2020). In contrast, SARS-CoV-2 can infect a wide range of human cell types as the symptoms of COVID-19 involve several different organ systems. The spike subunit of SARS-CoV-2 binds human angiotensin-converting enzyme two receptor (ACE2) and is primed by the cellular serine proteases TMPRSS2 and TMPRSS4 so that the virus can enter other host cells (Hoffmann et al., 2020). ACE2 is expressed in the entire human respiratory system, brain endothelium, and vascular smooth muscle cells (Hamming et al., 2004; Paniz-Mondolfi et al., 2020). Moreover, ACE2 and TMPRSS2 are expressed in esophageal keratinocytes, renal proximal tubules, pancreatic β-cells, and gastrointestinal epithelial cells (Gupta et al., 2020; Paniz-Mondolfi et al., 2020; Puelles et al., 2020; Qi et al., 2020). These facts are consistent with the observation that in certain post-acute sequelae of patients with COVID-19, SARS-CoV-2 maintains chronic symptoms by persisting in certain sites or tissue reservoirs after acute infection (Proal and VanElzakker, 2021). Possibly related to those, the numbers of CD1c^+^ myeloid and plasmacytoid DCs remained low even seven months after SARS-CoV-2 infection whether or not the patients were previously hospitalized (Pérez-Gómez et al., 2021). Our model reproduced long-term DC count reduction and showed that ongoing DC-induced inflammation was attributed to persistent viral infection that the host could not remove. The model simulations also predicted that successful elimination of the virus depends on the capacity of the host immune response which is directly related to viral load.

## Results

### Baseline model solution for immune response to SARS-CoV-2 infection

**Figure 2:**
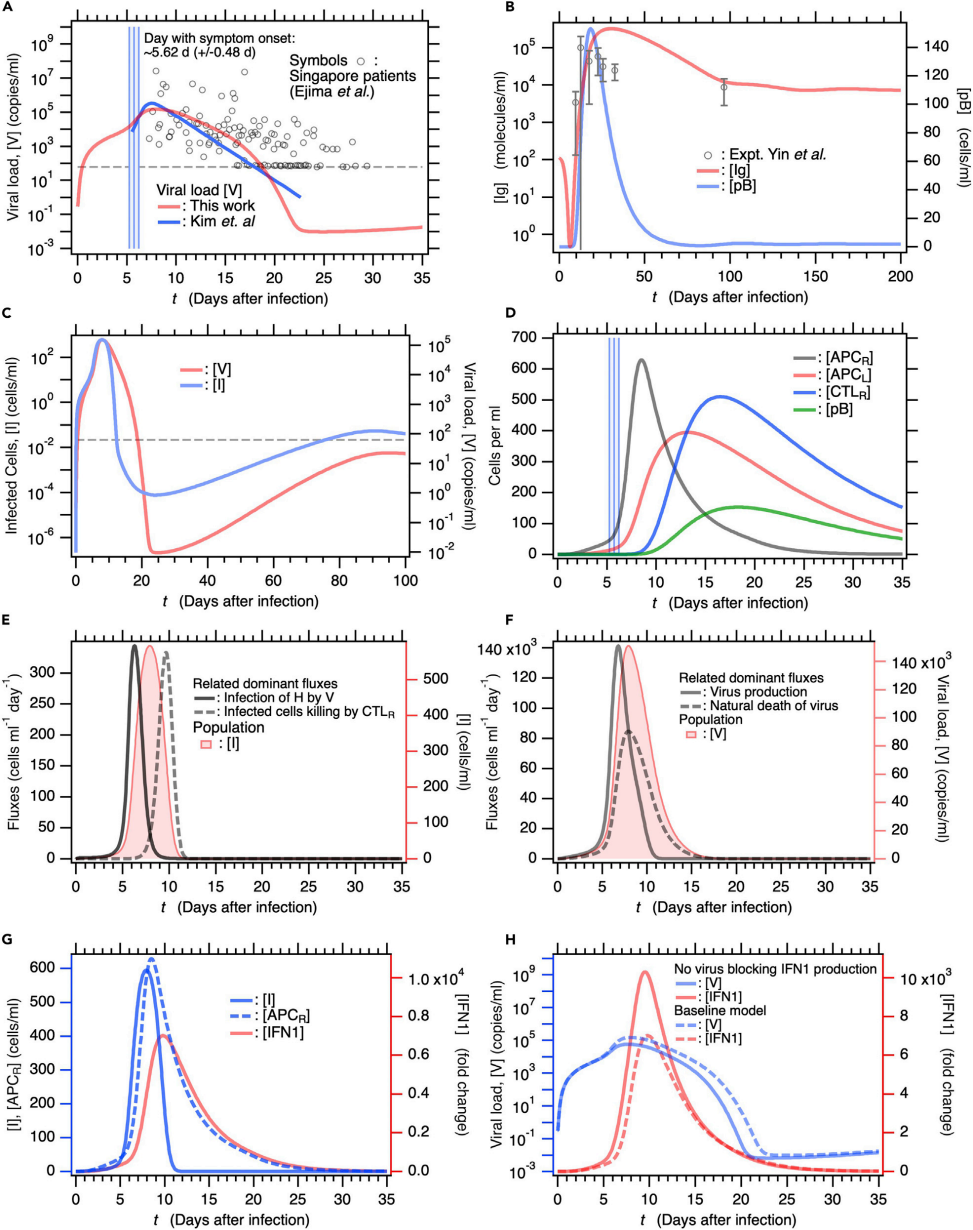
Baseline model solution for immune response as the function of number of days after SARS-CoV-2 infection (A) Comparison of baseline model solution for viral load [V] calculated from our mathematical model against [V] that Kim et al. determined by fitting a target cell-limited model to viral load data (Kim et al., 2021). Symbols are viral load data for Singapore patients with COVID-19 (Ejima et al., 2021; Young et al., 2020). Three vertical blue lines represent days of symptom onset (5.62 days: center line) and error (+/− 0.48 days: two lines beside it) which Ejima et al. estimated by applying a mathematical method to viral load data (Ejima et al., 2021). Dashed horizontal line indicates the viral detection limit. (B) Comparison of [Ig] obtained against longitudinally observed clinical data (Yin et al., 2020). Plasma B cell concentration [pB] is also shown (right axis). Standard deviation is indicated as error bar for the clinical data. (C) Long-term time course of infected cell [I] concentration (left axis) and [V] (right axis). Dashed horizontal line is the same as that in (a). (D) Time courses of concentrations of immunocytes [APC_R_], [APC_L_], [CTL_R_], and [pB]. Vertical blue lines are same as those in (a). (E) Fluxes contributing to variation in [I], namely, viral H infection and I killing by cytotoxic T lymphocytes (left axis). For comparison, [I] time course is also shown (right axis). (F) Fluxes contributing to variation in [V], namely, virus production by I and natural V degradation, are shown (left axis). Time course of [V] is also shown (right axis). (G) Time courses of [I] and [APC_R_] are shown (left axis). Both secrete IFN1 (right axis). (H) Time courses of [V] (left axis) and [IFN1] (right axis) for a model solution without mechanisms of evading IFN1 secretion are compared against those of baseline model (broken lines).

**Figure 3:**
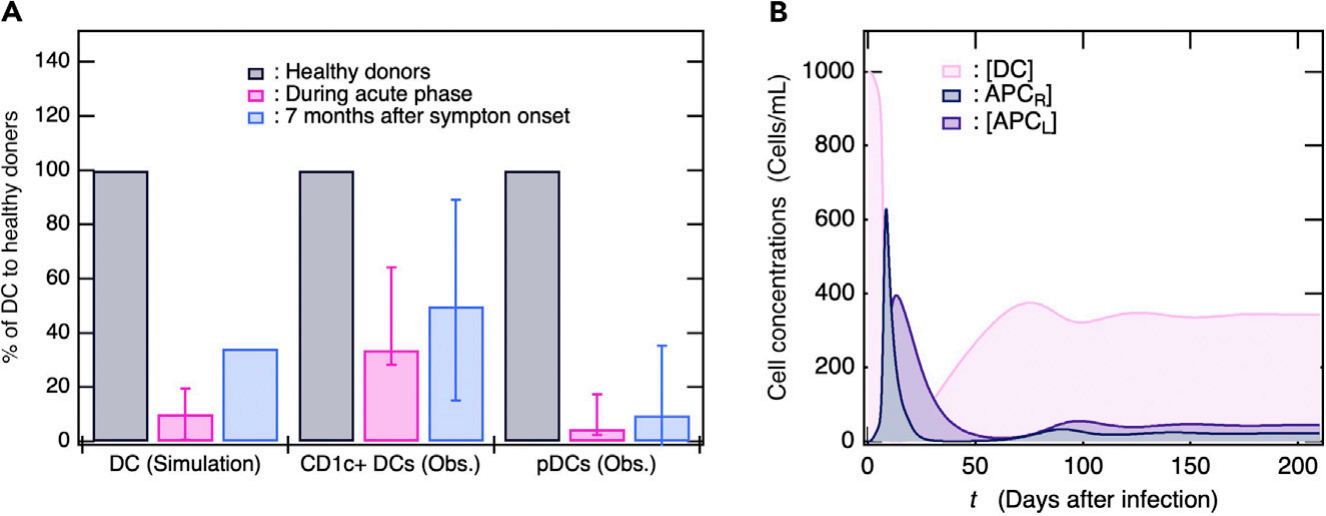
Substantial decrease in DC level in response to SARS-CoV-2 infection (A) Bar graphs represent % DC during the acute infection phase and 7 months after symptom onset compared with % DC in healthy donors. Clinical observation data were derived from literature (Pérez-Gómez et al., 2021). (B) Time courses of [DC], [APC_R_], and [APC_L_] obtained from simulation. In (a), the simulation DC values during the acute phase were taken at 3ays and 14 days after symptom onset in the same manner as clinical trials (Pérez-Gómez et al., 2021) and averaged. The bar is shown along with max/min values. For clinical data, % DC at 7 months after symptom onset is average for non-hospitalized and previously hospitalized patients.

Ordinary differential Equations 1, 2, 3, 4, 5, 6, 7, 8, 9, 10, 11, 12, 13, 14, 15, and 16 were solved using the initial values of the variables (STAR Methods, Table 1) and the parameters provided in the STAR Methods, Table 2. Figure 2 shows the variables for the baseline model solution plotted as a function of the number of days after infection. The viral load [V] was compared against the model solution (blue line) which Kim et al. determined using available viral load data (Kim et al., 2021) in addition to the data for Singapore patients with COVID-19 (Ejima et al., 2021; Young et al., 2020) (Figure 2A). The time to symptom onset after infection (∼5.62 days) and the error (± 0.48 days) have been mathematically determined by Ejima et al. using viral load data (Ejima et al., 2021), and those are denoted as the three vertical lines in Figure 2A. The peak viral load of [V] was attained at ∼2 days after symptom onset as previously reported by Kim et al. (2021). Figure 2B shows that the immunoglobulin concentration [Ig] time course is consistent with clinically observed data (Yin et al., 2020). The [Ig] increased in response to plasma B cell activation and Ig secretion as seen in Figure 2B. The correlation between infected cell [I] and viral load [V] is shown in Figure 2C. After [V] declined to a minimum, it gradually increased toward its steady-state as [I] also increased. The validity of our prediction regarding persistent SARS-CoV-2 infection will be addressed in terms of longitudinal observations of the DC immune response (Pérez-Gómez et al., 2021) (Figure 3) related to long COVID or post-acute sequelae of COVID-19 (PASC) (Proal and VanElzakker, 2021).

**Table 1:** Definition of the variables in the model

Symbol	Definition	Initial value
_[*H*]_	Population of susceptible healthy cells.	4.0 × 10^5^ cells ml^−1^
_[*I*]_	Population of infected cells.	0 cells ml^−1^
_[*DC*]_	Population of dendritic cells.	1.0 × 10^3^ cells ml^−1^ (Lee et al., 2009)
_[*APCR*]_	Population of antigen-presenting cells at respiratory tracts.	0 cells ml^−1^
_[*V*]_	The viral load of free SARS-CoV-2.	0.31 copies ml^−1^ (Hernandez-Vargas and Velasco-Hernandez, 2020)
_[*APCL*]_	Population of antigen-presenting cells at lymph nodes.	0 cells ml^−1^
_[*CD*__4_^+^__*T0*]_]_	Population of naive CD4^+^ T cells.	1.0 × 10^3^ cells ml^−1^ (Lee et al., 2009)
_[*Th1*]_	Population of type I helper T cells.	0 cells ml^−1^
_[*CD*8_+_*T0*]_	Population of naive CD8^+^ T cells.	1.0 × 10^3^ cells ml^−1^ (Lee et al., 2009)
_[*CTLL*]_	Population of cytotoxic T lymphocyte at lymph nodes.	0 cells ml^−1^
_[*CTLR*]_	Population of cytotoxic T lymphocyte at respiratory tracts.	0 cells ml^−1^
_[*Tfh*]_	Population of follicular helper T cells.	0 cells ml^−1^
_[*B0*]_	Population of naive B cells	1.0 × 10^3^ cells ml^−1^ (Lee et al., 2009)
_[*pB*]_	Population of plasma B cells.	0 cells ml^−1^
_[*Ig*]_	The fold change in immunoglobulin. This includes antiviral antibodies acting against SARS-CoV-2 that have been acquired upon seasonal human coronavirus infections as well as specific antiviral antibodies that are produced after SARS-CoV-2 infection, because a cohort of SARS-CoV-2–uninfected individuals showed to possess antiviral antibodies against SARS-CoV-2 (Ng et al., 2020).	110 molecules ml^−1^ (Lee et al., 2009)
_[*INF1*]_	The fold change in type-I interferon.	0 fold change

**Table 2:** Definition of the parameters in the model

Symbol	Definition	Values	Ref.
*λ*_*H*_	Supply rate of susceptible healthy epithelial cells.	4.0 × 10^3^ cells ml^−1^ day^−1^	
*δ*_*H*_	Natural death rate of susceptible healthy epithelial cells.	1.0 × 10^−2^ day^−1^	
*λ*_*DC*_	Supply rate of susceptible healthy cells.	1.0 × 10^1^ cells ml^−1^ day^−1^	
*δ*_*DC*_	Natural death rate of healthy cells.	1.0 × 10^−2^ day^−1^	
*λ*_*CD*4_	Supply rate of susceptible healthy cells.	2.0 × 10^1^ cells ml^−1^ day^−1^	
*δ*_*CD*4_	Natural death rate of healthy cells.	2.0 × 10^−2^ day^−1^	(Oprea and Perelson, 1996; Vonboehmer and Hafen, 1993)
*λ*_*CD*8_	Supply rate of susceptible healthy cells.	2.0 × 10^1^ cells ml^−1^ day^−1^	
*δ*_*CD*8_	Natural death rate of healthy cells.	2.0 × 10^−2^ day^−1^	(Oprea and Perelson, 1996; Vonboehmer and Hafen, 1993)
*λ*_*B*_	Supply rate of susceptible healthy cells.	2.0 × 10^2^ cells ml^−1^ day^−1^	
*δ*_*B*_	Natural death rate of healthy cells.	2.0 × 10^−1^ day^−1^	(Chan and Maclennan, 1993; Oprea and Perelson, 1996)
*π*_*I*_	Infection rate of susceptible healthy epithelial cells	2.0 × 10^−6^ day^−1^ mL copies^−1^	
*β*_*I*_	Antibody neutralization rate	5.0 × 10^−3^ mL molecules^−1^	
*δ*_*I*_	Natural death rate of infected cells.	1.0 × 10^−2^ day^−1^	*δ*_*I*_ is much smaller than *δ*_*V*_. (Kim et al., 2021)
*k*_*I*_	Rate of killing of infected cells by cytotoxic T lymphocyte at respiratory tracts	2.0 × 10^−2^ day^−1^ mL cells^−1^	
*π*_*APC*_	Infection and antigen-presenting rate of dendritic cells	2.0 × 10^−6^ day^−1^ mL copies^−1^	
*α*_*recruit*_	Recruitment efficiency of dendritic cells by type-I interferon	1.0 × 10^−3^ (fold change)^−1^	
*α*_*APC*_	Regulation of antigen-presenting rate of dendritic cells by Ig	3.0 × 10^−2^ mL molecules^−1^	
	Natural death rate of antigen-presenting cells in respiratory tracts	0.1 day^−1^	
*μ*_*APC*_	Migration rate of antigen-presenting cells from respiratory tracts to lymph nodes	0.2 days^−1^	
*π*_*V*_	Production rate of virus from infected cells	700 days^−1^ copies cells^−1^	
*β*_*V*_	Inhibition rate of virus production by type-I interferon	1.0 × 10^−3^ (fold change)^−1^	
*δ*_*V*_	Clearance rare of virus	0.56 days^−1^	(Ejima et al., 2021)
*γ*_*Ig*_	Neutralized rate of virus by antibodies	9.0 × 10^−6^ day^−1^ mL molecules^−1^	
	Natural death rate of antigen-presenting cells at lymph nodes	0.1 day^−1^	
*π*_*Th*1_	Differentiation rate of naive CD4^+^ T cells into type I helper T cells	6.0 × 10^−6^ day^−1^ mL cells^−1^	
*α*_*Th*1_	Regulation of CD4^+^ T cell differentiation rate into Th1 cells by type I interferon	1.0 × 10^−4^ (fold change)^−1^	
*π*_*Tfh*_	Differentiation rate of naive CD4^+^ T cells into follicular helper T cells	5.0 × 10^−5^ day^−1^ mL cells^−1^	
*α*_*Tfh*_	Regulation of CD4^+^ T cell differentiation rate into Tfh cells by type I interferon	5.0 × 10^−4^ (fold change)^−1^	
*δ*_*Th*1_	Natural death rate of Th1 cells	0.4 days^−1^	(Lee et al., 2009)
*δ*_*Tf*h_	Natural death rate of Tfh cells	0.4 days^−1^	(Lee et al., 2009)
*π*_*CTL*_	Generation rate of cytotoxic T lymphocyte from naive CD8^+^ T cells	1.0 × 10^−4^ day^−1^ mL^2^ cells^−2^	
*δ*_*CTL*_	Natural death rate of CTL cells	0.1 day^−1^	(Zarnitsyna et al., 2016)
*μ*_*CTL*_	Migration rate of CTL cells from lymph nodes to respiratory tracts	1.2 days^−1^	(Zarnitsyna et al., 2016)
*π*_*pB*_	Generation rate of plasma B cells from naive B cells	8.0 × 10^−7^ day^−1^ mL^2^ cells^−2^	
*δ*_*pB*_	Natural death rate of plasma B cells	0.1 day^−1^	(Lee et al., 2009)
*π*_*Ig*_	Antibody production rate.	3.0 × 10^2^ day^−1^ molecules ml^−1^ cells^−1^	
*δ*_*Ig*_	Degradation rate of antibody	0.07 days^−1^	(Nikin-Beers and Ciupe, 2015)
*ξ*_*Ig*_	Consumption rate of Ig upon Ig-binding to virus	*N*_*S*_ × *γ*_*Ig*_ = 2.16 × 10^−4^ day^−1^ mL copies^−1^	*N*_*S*_ = 24 (Ke et al., 2020)
σ_1_	Secretion rate of type I interferon by infected cells	0.01 days^−1^ (fold change) ml cells^−1^	
*σ*_*APC*_	Secretion rate of type I interferon by APC cells	10 days^−1^ (fold change) ml cells^−1^	(Ghosh, 2021)
*δ*_*IFN*1_	Degradation rate of type I interferon	0.7 days^−1^	(Ghosh, 2021; Harari et al., 2014)

Figure 2D shows the time course of activation of innate and acquired immunocytes after the viral infection. Transformation of DC into antigen-presenting cells (APC_R_) at the infection sites begins at the onset of the viral infection and rapidly increases after symptom onset (vertical blue lines). APC_R_ migrates from the infection site to a lymph node (Figure 1) and APC_L_ increases (Figure 2D). APC_L_ induces naive CD4^+^ T cells to differentiate into type-I helper T cells (Th1) and T follicular helper cells (Tfh). Thence, APC_L_ and Th1 induce naive CD8^+^ T cells (CD8^+^T_0_) to differentiate into cytotoxic T lymphocytes (CTL_L_). From there, the CTL_L_ migrate to the infection sites (Figure 1) and [CTL_R_] increases. Similarly, APC_L_ and Tfh induce naive B cells (B_0_) to differentiate into plasma B cells (pB) (Figure 1) and [pB] increases.

Figures 2E and 2F show the dominant fluxes that explain the observed variations in [I] and [V], respectively. Viral infection in healthy epithelial cells increases [I]. Thereafter, CTL_R_ kill I and cause [I] to decline sharply (Figure 2E). The number of viral particles that have been replicated by infected cells within the host is reduced by natural viral degradation. However, this process is comparatively slow (Figure 2F). Figure 2G shows the time course for [I] and [APC_R_] involved in IFN1 production. [I] increased faster than [APC_R_], whereas the increases and decreases in [IFN1] were synchronized with the changes in [APC_R_] rather than [I]. In fact, the IFN1 production rate used for the baseline model is equal to 1,000-fold lower in infected cells than APC_R_ (STAR Methods, Table 2). The reason for using these parameters is that experimental studies demonstrate that SARS-CoV-2 possesses several mechanisms to evade the IFN1-mediated immune response (Sa Ribero et al., 2020). However, if a virus lacked these mechanisms (STAR Methods, Table 3), it can be more rapidly cleared from the host as shown by the blue solid line in Figure 2H.

**Table 3:** The parameter used for the model with no blocking production of IFN1 by SARS-CoV-2

Symbol	Definition	Values	Ref.
_*σ*1_	1000 times increased.	10 days^−1^ (fold change) ml cells^−1^	Comparable with σ_*APC*_

### Dendritic cell deficiency persists for >7 months after SARS-CoV-2 infection

DCs play key roles in defending against viral infections. When DCs capture viruses, these functions as APCs, and one of the subsets, plasmacytoid DCs (pDCs), produces abundant IFN1. Whether or not patients with COVID-19 were previously hospitalized, the numbers of their CD1c^+^ myeloid DCs and pDCs were lower than those for healthy donors during the acute infection phase and even 7 months after the initial SARS-CoV-2 infection (Figure 3A) (Pérez-Gómez et al., 2021). Hence, the DCs induce and partially sustain ongoing inflammation that can be related to long COVID or PASC (Proal and VanElzakker, 2021). A multisystem inflammatory syndrome in children (MIS-C) had been recognized (Gruber et al., 2020), providing evidence of long-term DC deficiency. Patients with MIS-C had SARS-CoV-2 exposure, mounted an antibody response with similar neutralization capability, and had lower pDC levels than the healthy group. In addition, the levels of non-classical monocytes and a subset of natural killer cells were also reduced in the MIS-C group, thus demonstrating the relationship of these cell populations with the ongoing inflammation in the child participants (Gruber et al., 2020). Our baseline simulation predicted that the number of DCs rapidly decreases during the acute phase and increases thereafter but is nonetheless lower than it was before the infection (Figure 3B). The simulated proportions of DC reduction in infected patients compared with healthy donors were consistent with those determined by clinical observation (Figure 3A) (Pérez-Gómez et al., 2021). Figure 2C shows that [V] increases after decreasing to a minimum possibly because certain viruses cannot be removed by the host as a consequence of persistent infection. In our baseline simulation, long-term DC deficiency was attributable to persistent SARS-CoV-2 infection, where viral load was often below the detection limit. The simulation result was consistent with the clinical observation that both short-term and long-term COVID-19 symptoms were commonly associated with persistent DC deficiency (Pérez-Gómez et al., 2021).

### Deficient immune responses might cause severe COVID-19

Attenuation of the immune system during aging is associated with increased susceptibility to various infectious diseases, a decrease in the ability to fight new infections, re-emergence of latent infections, and increases in disease severity. Older people are at a much higher risk of developing severe or fatal COVID-19 than younger people (Sette and Crotty, 2021). We examined the effects of aging on the immune system to understand the risk factors and typical mechanisms related to COVID-19 exacerbation. There are no major differences between young and elderly subjects in terms of the numbers or phenotypes of their DC subsets. In contrast, the ability of DCs to phagocytose antigens, migrate, and prime T cell responses declines with advancing age (Agrawal and Gupta, 2011; Sridharan et al., 2011). Thus, we explored how reductions in the following DC functions affect COVID-19 severity: (1) DC transformation into APC, (2) APC migration toward lymph nodes, (3) and (4) CD4^+^T_0_ differentiation into Th1 and Tfh by APC_L_, (5) CD8^+^T_0_ differentiation into CTL_L_ by APC_L_ and Th1, (6) B_0_ differentiation into pB by APC_L_ and Th1, and (7) IFN1 production by APC_R_ (STAR Methods, Table 4, Models one and 2).

**Table 4:** The parameters used in severe symptom models by risk factors of aging

Model 1. 90% activity of APCs		
*π*_*APC*_	10% reduced.	1.8 × 10^−6^ day^−1^ mL copies^−1^
*μ*_*APC*_	10% reduced.	0.18 days^−1^
*π*_*Th*1_	10% reduced.	5.4 × 10^−6^ day^−1^ mL cells^−1^
*π*_*Tfh*_	10% reduced.	4.5 × 10^−5^ day^−1^ mL cells^−1^
*π*_*CTL*_	10% reduced.	0.9 × 10^−4^ day^−1^ mL^2^ cells^−2^
*π*_*pB*_	10% reduced.	7.2 × 10^−7^ day^−1^ mL^2^ cells^−2^
*σ*_*APC*_	10% reduced.	9 days^−1^ (fold change) ml cells^−1^

**Model 2. 80% activity of APCs**		

*π*_*APC*_	20% reduced.	1.6 × 10^−6^ day^−1^ mL copies^−1^
*μ*_*APC*_	20% reduced.	0.16 days^−1^
*π*_*Th*1_	20% reduced.	4.8 × 10^−6^ day^−1^ mL cells^−1^
*π*_*Tfh*_	20% reduced.	4.0 × 10^−5^ day^−1^ mL cells^−1^
*π*_*CTL*_	20% reduced.	0.8 × 10^−4^ day^−1^ mL^2^ cells^−2^
*π*_*pB*_	20% reduced.	6.4 × 10^−7^ day^−1^ mL^2^ cells^−2^
*σ*_*APC*_	20% reduced.	8 days^−1^ (fold change) ml cells^−1^

**Model 3. 50% reduction in IFN1-signaling effects by autoantibodies**		

*β*_*V*_	50% reduced	5.0 × 10^−4^ (fold change)^−1^
*α*_*recruit*_	50% reduced	5.0 × 10^−4^ (fold change)^−1^
*α*_*Th*1_	50% reduced	5.0 × 10^−5^ (fold change)^−1^
*α*_*Tfh*_	50% reduced	2.5 × 10^−4^ (fold change)^−1^

**Model 4. 100% reduction in IFN1-signaling effects by autoantibodies**		

*β*_*V*_	100% reduced	0 (fold change)^−1^
*α*_*recruit*_	100% reduced	0 (fold change)^−1^
_*Th*1_	100% reduced	0 (fold change)^−1^
*α*_*Tfh*_	100% reduced	0 (fold change)^−1^

**Model 5. 10% concentration of naive CD8^+^T**_**0**_**cell**		

*λ*_*CD*8_	0.1 times increased	2.0 cells ml^−1^ day^−1^
[*CD*8 + *T*_*o*_]	0.1 times increased	1.0 × 10^2^ cells ml^−1^

Congenital and acquired defects in IFN1 signaling can result in severe COVID-19. IFN1 autoantibodies were detected in plasma samples from a large cohort comprising patients with COVID-19 and pre-pandemic controls (Bastard et al., 2021). The incidence of IFN1-neutralizing autoantibodies increasing was particularly evident in subjects >70 years old in the control cohort. Hence, the autoantibodies targeting IFN1 have manifested a common form of acquired immunodeficiency associated with ∼20% of all COVID-19 fatalities (Bastard et al., 2021). In the present study, we investigated how reductions in IFN1 signaling affect (1) the suppression of viral replication, (2) the migration of APC_R_ and CTL_L_, (3) and (4) the differentiation of CD4^+^T_0_ into Th1 and Tfh, and consequently the severity of COVID-19 (STAR Methods, Table 4, Model 3, 4). With advancing age, the number of naive CD8^+^ T cells decreases by approximately one order of magnitude, whereas the numbers of naive CD4^+^ T and B cells do not (Westera et al., 2015). Thus, we also examined how age-related reductions in [CD8^+^T_0_] affect the viral load time course (STAR Methods, Table 4, Model 5).

**Figure 4:**
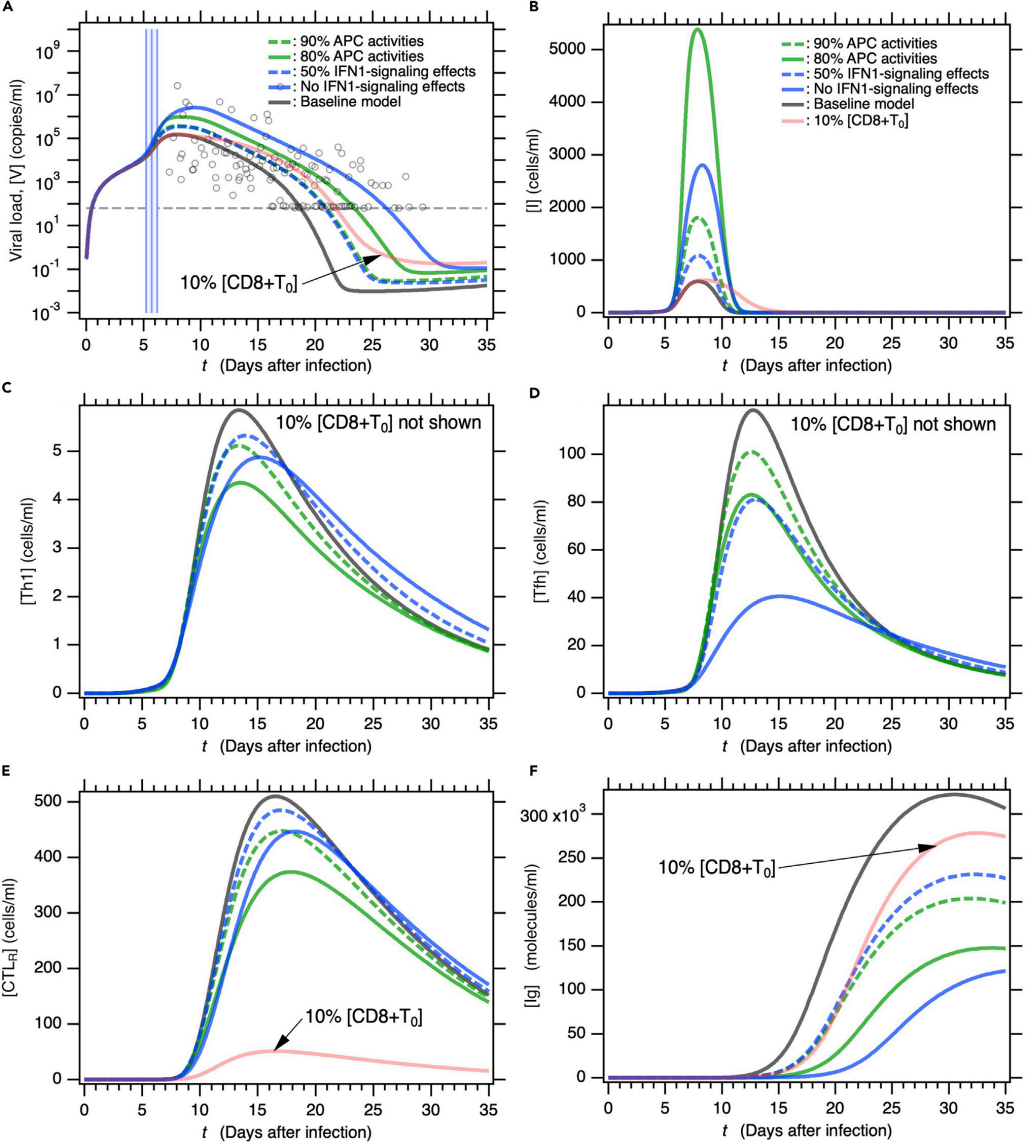
Model solutions for severe COVID-19 symptoms depending on typical age-related risk factors (A–F) Time courses of (A) viral load [V], (B) infected cells [I], (C) type-I helper T cells [Th1], (D) T follicular helper cells [Tfh], (E) cytotoxic T lymphocytes at infection sites [CTL_R_], and (F) immunoglobulin [Ig] in patients with various age-related risk factors. Five age-related risk factors, 90% APC activity (model 1), 80% APC activity (model 2), 50% reduction in IFN1 signaling (model 3), no IFN1 signaling (model 4), and 10% [CD8^+^T_0_] (model 5) were examined. Parameters used in these models are listed in the STAR MethodsTable 4.

Reduced APC activity and deficient IFN1 signaling increase [V] immediately after symptom onset and also contribute to higher long-term viral loads (Figure 4A). The increase in [V] following symptom onset is related to an increase in [I] (Figure 4B). In contrast, a decrease in [CD8^+^T_0_] by one order of magnitude does not affect [V] immediately after symptom onset but does retard virus removal (Figure 4A). In this case, the lack of any increase in the maximum [V] is attributed mainly to the lack of increase in the maximum [I] (Figure 4B). Prolonged infection caused by a dramatic reduction in [CTL_R_] might account for the observed delays in viral clearance despite moderate maximum viral loads (Figures 4B and 4E, red line). Impairment of APC activity and IFN1 signaling ordinarily affects naive CD4^+^ T cell priming into Th1 and Tfh (Figures 4C and 4D), thereby lowering [CTL_R_] and [Ig] (Figures 4E and 4F). As the decrease in [CD8^+^T_0_] affects neither [Th1] nor [Tfh], it was omitted from Figures 4C and 4D. In response to deficient APC activity and neutralization of IFN1 signaling, maximum [I] is higher for the former than the latter, maximum [V] is higher for the latter than the former. Therefore, these results reflect that IFN1 signaling profoundly affects the suppression of viral replication in infected cells. [V]s for the 90% APC activities and the 50% IFN1 signaling effects overlap each other by chance (Figure 4A), whereas the other variables do not (Figures 4B–4F). The higher maximum in [I] for the 80% APC activities (Figure 4B) was attributed to a raise in viral infection flux that was indirectly mediated by the accumulated effects of reduction in each reaction rate related to APC activities (Figure S2). Taken together, the foregoing findings indicate that all patients that are partially deficient in innate and/or acquired immunity because of inflammation and (immune) disease are also potentially at high risk of severe or even fatal COVID-19.

### The probability of complete SARS-CoV-2 elimination increases with the ability of the immune system to suppress viral replication

**Figure 5:**
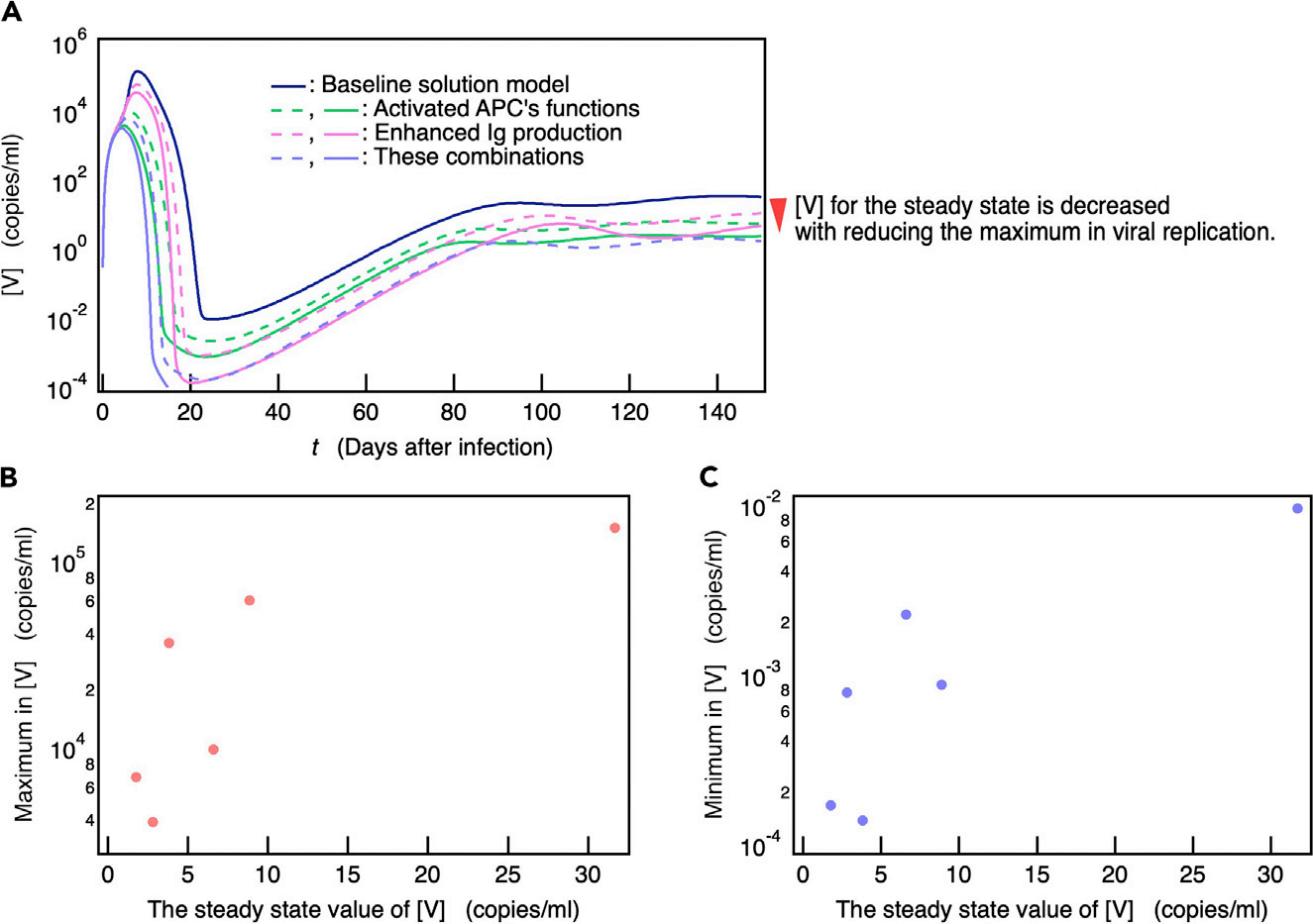
Ability of the immune response to suppress viral replication is necessary for complete SARS-CoV-2 clearance (A) Time courses of [V] in six models with several fold increases in parameters related to APCs function and/or Ig production (see the STAR MethodsTable 5). (B and C) The maximum and (C) minimum found in [V] for each model are plotted as a function of its steady-state [V]. In models of the highest immune capacity, viral load becomes very low ([V] < ∼10^−4^), virus is assumed to be completely eliminated from the host, and time evolution is discontinued in (A). Hence, [V] for this scenario is not shown in (B) and (C). In (A), a colored solid line represents stronger immune response than a colored dashed line.

The baseline simulation (Figure 2) indicates that numerous patients cannot successfully remove SARS-CoV-2. Furthermore, the linear stable analysis demonstrates that a steady-state with zero viral load is unstable, i.e., the baseline model never reaches the steady-state with zero viral load (Table S1). However, if the viral infection rate *π*_*I*_ and the production rate of virus *π*_*V*_ were reduced from these values of the baseline model, a steady-state with zero value of [V] was confirmed to be stable (Table S2). In contrast, a steady-state with a finite [V] value in the baseline model was asymptotically stable (Table S3). Therefore, the persistent viral infection can be ongoing within the host and might cause long COVID or PASC. To evaluate this prediction, we investigated the relationships between [V] after infinite time (steady-state [V]) and the model parameters. The model parameters significantly reducing the steady-state values of [V] and [I] were disclosed by a steady-state sensitivity analysis (Bergmann et al., 2017) (Table S4). The most sensitive parameters to a decrease in [V] were associated with Ig production (*π*_*Ig*_, *π*_*pB*_, and *π*_*Tfh*_) and APC activation (*π*_*APC*_, *α*_*APC*_, and *μ*_*APC*_) except for parameters related to steady-state concentration of immune cells before viral infection (Table S4). The [V] time courses for six models with several fold increases in the Ig production and/or APC activation parameters (STAR Methods, Table 5) are shown in Figure 5A along with the baseline model. When [V] < 10^−4^, time evolution discontinued and it was assumed that the virus in the model with the highest immune capacity was entirely eliminated from the host. Figure 5A shows that long-term [V] and also maximum [V] decreased with increasing parameter values, particularly immune ability. Hence, the maximum [V] asymptotically decreased along with the decrease in steady-state [V] (Figure 5B). The [V] time courses in these immune-enhanced models deviated from the clinical observation data shown in Figure 2A. These findings suggest that patients cannot completely remove even average SARS-CoV-2 loads that are undergoing replication.

**Table 5:** The parameters used to reduce the steady-state values of [V] and [I]

Model 1. Two times activated APCs		
*π*_*APC*_	× 2	4.0 × 10^−6^ day^−1^ mL copies^−1^
*α*_*APC*_	× 2	6.0 × 10^−2^ mL molecules^−1^
*μ*_*APC*_	× 2	0.4 days^−1^

**Model 2. Three times activated APCs**		

*π*_*APC*_	× 3	6.0 × 10^−6^ day^−1^ mL copies^−1^
*α*_*APC*_	× 3	9.0 × 10^−2^ mL molecules^−1^
*μ*_*APC*_	× 3	0.6 days^−1^

**Model 3. 1.5 times enhanced production of Ig**		

*π*_*Ig*_	× 1.5	4.5 × 10^2^ day^−1^ molecules ml^−1^ cells^−1^
*π*_*pB*_	× 1.5	12.0 × 10^−7^ day^−1^ mL^2^ cells^−2^
*π*_*Tfh*_	× 1.5	7.5 × 10^−5^ day^−1^ mL cells^−1^

**Model 4. Two times enhanced production of Ig**		

*π*_*Ig*_	× 2	6.0 × 10^2^ day^−1^ molecules ml^−1^ cells^−1^
*π*_*pB*_	× 2	16.0 × 10^−7^ day^−1^ mL^2^ cells^−2^
*π*_*Tfh*_	× 2	10.0 × 10^−5^ day^−1^ mL cells^−1^

**Model 5. Two times activated APCs and 1.5 times enhanced production of Ig**		

*π*_*APC*_	× 2	4.0 × 10^−6^ day^−1^ mL copies^−1^
*α*_*APC*_	× 2	6.0 × 10^−2^ mL molecules^−1^
*μ*_*APC*_	× 2	0.4 days^−1^
*π*_*Ig*_	× 1.5	4.5 × 10^2^ day^−1^ molecules ml^−1^ cells^−1^
*π*_*pB*_	× 1.5	12.0 × 10^−7^ day^−1^ mL^2^ cells^−2^
*π*_*Tfh*_	× 1.5	7.5 × 10^−5^ day^−1^ mL cells^−1^

**Model 6. Three times activated APCs and 1,5 times enhanced production of Ig**		

*π*_*APC*_	× 3	6.0 × 10^−6^ day^−1^ mL copies^−1^
*α*_*APC*_	× 3	9.0 × 10^−2^ mL molecules^−1^
*μ*_*APC*_	× 3	0.6 days^−1^
*π*_*Ig*_	× 1.5	4.5 × 10^2^ day^−1^ molecules ml^−1^ cells^−1^
*π*_*pB*_	× 1.5	12.0 × 10^−7^ day^−1^ mL^2^ cells^−2^
*π*_*Tfh*_	× 1.5	7.5 × 10^−5^ day^−1^ mL cells^−1^

Figure 5A also shows that the minimum [V] decreased with increasing those parameter values, namely, the immune ability. Therefore, as the steady-state [V] decreases, the minimum [V] becomes so small that it is effectively zero (Figure 5C). In this case, the patient is virtually cured as [V] and [I] should stochastically converge to zero. Therefore, for instance, when Ig production and APC activity are sufficiently high, the patient is asymptomatic, if viral replication is suppressed, the virus may have been successfully removed, and the patient is cured. Otherwise, viral infection remains in the host and can progress into long COVID or PASC (Proal and VanElzakker, 2021).

## Discussion

SARS-CoV-2 can reach and infect the cells in multiple organs and tissues via hematogenous diffusion from heavily infected airways and lungs (Proal and VanElzakker, 2021). Influenza A virus causes a self-limited acute viral infection in the upper respiratory tract (Miyazawa, 2020). In contrast, ACE2 and TMPRSS2 enable SARS-CoV-2 to infect and penetrate a wide range of host cell types (A. Gupta et al., 2020; Paniz-Mondolfi et al., 2020; Puelles et al., 2020; Qi et al., 2020). Evidence for systemic SARS-CoV-2 infection was provided from the complete autopsies of 44 patients with COVID-19 and demonstrated SARS-CoV-2 distribution, replication, and cell-type specificity throughout the human body. The virus was widely distributed even in deceased patients with asymptomatic to mild COVID-19 (Chertow et al., 2021). In fact, viral replication was detected in multiple extrapulmonary tissues and systemic infection persisted for more than several months.

PASC is being diagnosed in patients with severe acute COVID-19 as well as those with only mild or even no symptoms (Logue et al., 2021). The long-term symptoms observed in patients with PASC may be the consequences of organ and tissue injury caused by SARS-CoV-2 and/or coagulation and inflammation during acute COVID-19 (Del Rio et al., 2020). In contrast, SARS-CoV-2 may remain within certain patients with PASC, thereby causing chronic inflammation and dysfunction in certain organs and tissues. Several studies reported that patients infected with SARS-CoV-2 may not fully clear it for very long periods of time (L. Huang et al., 2021; Liotti et al., 2021; Vibholm et al., 2021). In a trial on 203 post-symptomatic participants with previous RT-PCR-verified SARS-CoV-2 infection, 5.3% of the subjects remained virus-positive even 90 days after recovery (Vibholm et al., 2021). There were no differences between PCR-positive and PCR-negative subjects in terms of SARS-CoV-2-specific Ig. However, the PCR-positive group presented with significantly stronger SARS-CoV-2-specific CD8^+^T cell responses (Vibholm et al., 2021).

Dendritic cells (DCs) are components of innate immunity and play key roles in the host SARS-CoV-2 response. Recovery of DC defects after COVID-19 is vital as the normalization of the innate immune system after acute insults are required for appropriate responses to new microbial challenges. However, patients with acute SARS-CoV-2 infection present with substantially reduced DC counts that might not normalize even 7 months after the initial acute SARS-CoV-2 infection (Pérez-Gómez et al., 2021) (Figure 3). The observed long-term decrease in DC number may be explained by the migration of DC cells to inflammatory sites caused by persistent SARS-CoV-2 infection contributing to long COVID. As evidence supporting the persistent viral infection, in addition to long-term pDC deficient, reductions in non-classical monocytes and a subset of natural killer cells have been observed in the MIS-C group (Gruber et al., 2020).

Numerous studies have reported persistent single-strand RNA virus infections (Doi et al., 2016; Ireland et al., 2020; Randall and Griffin, 2017), especially in the CNS (Kristensson and Norrby, 1986). The CNS is considered a target for several different persistent viral infections as neurons are post-mitotic single cells that persist throughout the entire lifetime of the host. Thus, neurons may provide a more protective environment for long-term viral persistence than rapidly multiplying cells that can sequester microbial pathogens (Kristensson and Norrby, 1986). As the other well-known case, hepatitis C virus (HCV) also establishes persistent infection by evading the host innate immune response (Patra et al., 2019; Rehermann, 2009). These abundant clinical observations support the potential for the persistence of the SARS-CoV-2 infection to be ongoing in patients with PASC symptoms. In an earlier clinical trial, only ∼5% of all subjects were positive for SARS-CoV-2 according to RT-PCR nasopharyngeal testing ∼90 days after infection and there was no apparent transmission to close contacts (Vibholm et al., 2021). Nevertheless, there is no consensus that patients with persistent SARS-CoV-2 infection are not contagious. Persistent SARS-CoV-2 infection and infectivity merit further investigation so that treatments for PASC may be developed and the COVID-19 pandemic may be managed more effectively.

### Limitations of the study

Our mathematical model of the host immune response to SARS-CoV-2 demonstrated that age-related risk factors such as a decrease in innate immune cell activity and/or an increase in autoantibody-mediated IFN1 signaling inhibition markedly increased viral load. Our model also predicted persistent reductions in DC abundance and showed that patients with severe and even mild symptoms may develop long COVID-19 as they may not effectively eliminate the virus. However, the foregoing model did not regard memory T and B cells. Hence, the mechanisms by which these memory effects including owing to vaccination influence the immune response to SARS-CoV-2 infection and long COVID remain unknown and further investigations including longitudinal observations on prognosis and immune response of unvaccinated and vaccinated patients are essential. Our future studies using mathematical models extended to address these issues are important to further understanding.

## STAR★Methods

### Key resources table

**Table:** 

REAGENT or RESOURCE	SOURCE	IDENTIFIER
**Software and algorithms**		

COPASI biochemical system simulator (v. 4.28)	COPASI	https://copasi.org/
Igol Pro (v. 8.04)	WaveMetrics	https://www.wavemetrics.com/

### Resource availability

#### Lead contact

The COPASI input data used to generate the data for the current study are available from the corresponding author (TS) upon reasonable request.

#### Materials availability

This study did not generate new materials or reagents.

### Experimental model and subject details

#### Mathematical model

A mathematical model comprising ordinary differential equations (ODEs) was used to investigate host immune responses to SARS-CoV-2 infection. In this model, the immune responses were assumed to occur at the infection sites and the lymph nodes (Figure 1). The respiratory tract was assumed to be the main initial site of infection. However, SARS-CoV-2 can reach and infect the cells in multiple organs and tissues via hematogenous diffusion from heavily infected airways and lungs (Proal and VanElzakker, 2021). Therefore, all cells expressing ACE2 were presumed to be the target of infection by SARS-CoV-2 virus. The healthy epithelial cells with ACE2 were supplied at rate *λ*_*H*_ and underwent apoptosis at the rate *δ*_*H*_ (Equation 1). In the present study, it is assumed that the uninfected epithelial cells are generated to return to a certain number even if they are temporarily reduced due to viral infection. Thus, a model which simply satisfies this dynamic behavior under the balance between the supply and death (Equation 1) was employed. The dendritic cells (Equation 3), naive CD4^+^ cells (Equation 9), naive CD8^+^ T cells (Equation 11), and naive B cells (Equation 14) were assumed to regenerate and die in the same manner. The rate of infection of the target epithelial cells with free virus (*π*_*I*_[*H*][*V*]) was divided by (1+*β*_*I*_[*I*_*g*_][*V*]) (Equation 2). Thus, the infection was suppressed by the binding of Ig to the virus. Here, Ig was assumed to include antiviral antibodies acting against SARS-CoV-2 that have been acquired upon seasonal human coronavirus infections as well as specific antiviral antibodies that are produced during SARS-CoV-2 infection, because a cohort of SARS-CoV-2–uninfected individuals were identified to possess antiviral antibodies against SARS-CoV-2 (Ng et al., 2020). The dendritic cells that are recruited and activated by IFN1 (Fitzgerald-Bocarsly and Feng, 2007) efficiently capture antibody-neutralized virus (van Montfort et al., 2007) and transform into APC_R_ at the infection sites. Therefore, the rate of DC transformation into APC_R_ was given as *π*_*APC*_[*DC*][*V*] multiplied by (1+*α*_*recruit*_[*INF*1]) (1+*α*_*APC*_[*Ig*]) (Equation 4). Viral replication is inhibited by IFN1-induced genes (Makris et al., 2017; Sa Ribero et al., 2020). Hence, the viral replication rate was proportional to the inverse of (1+*β*_*V*_[*INF*1]) (Equation 5). In this equation, the virus neutralized by Ig was removed at a rate proportional to *γ*_*Ig*_[*Ig*]. The production rates of IFN1 by I and APC_R_ were *σ*_*I*_[*I*] and *σ*_*APC*_[*APC*_*R*_], respectively (Equation 6), and *σ*_*I*_ was assumed to be 1000-fold lower than *σ*_*APC*_ (Table 2) due to the several mechanisms employed by SARS-CoV-2 to evade the IFN1-mediated immune response (Sa Ribero et al., 2020).

APC_R_ was assumed to migrate into lymph nodes with the rate *μ*_*APC*_[*APC*_*R*_] (Equation 8). The development of naive CD4+T_0_ cells into Th1 and Tfh by APC_L_ (Swain et al., 2012) is stimulated by IFN1 (Cucak et al., 2009; Huber and Farrar, 2011). Therefore, the rates of CD4+T_0_ transformation into Th1 and Tfh, *π*_*Th*1_[*APC*_*L*_][CD4+*T*_*o*_] (Equation 10) and *π*_*Tfh*_[*APC*_*L*_][CD4+*T*_*o*_] (Equation 13), were assumed to be multiplied by (1+*α*_*Th*1_[*INF*1]) and (1+*α*_*Tfh*_[*INF*1]), respectively. APC_L_ and Th1 activate CD8+T_0_ cells, which then differentiate into CTL_L_ cells (Swain et al., 2012). Thus, the rate of CD8+T_0_ transformation was given as *π*_*CTL*_[*APC*_*L*_][*Th*1][CD8+*T*_*o*_] (Equation 12). CTL_L_ are recruited and activated by IFN1 (Huber and Farrar, 2011); therefore, the migration rate of CTL_L_ toward the sites of infection was assumed to be *μ*_*CTL*_(1+*α*_*recruit*_[*INF*1][*CTL*_*L*_]) (Equation 12).

APC_L_ and Tfh activate naive B_0_ cells, which differentiate into pB cells (Akkaya et al., 2020). Thus, the rate of B_0_ transformation was assumed to be *π*_*pB*_[*APC*_*L*_][*Tfh*][*B*_*o*_] (Equation 15). pB cells produced Ig with the rate *π*_*Ig*_[*pB*], and the Ig degradation rate was *δ*_*Ig*_[*Ig*] (Equation 16). In this equation, Ig was consumed upon binding to the virus at the rate *ξIg*[*Ig*][*V*]. The model ODEs are listed below:

##### Infection sites

(Equation 1) $$d\left[H\right]/dt=\lambda_{H}-\delta_{H}\left[H\right]-\pi_{I}\left[H\right]\left[V\right]/\left(1+\beta_{I}\left[Ig\right]\left[V\right]\right)\text{,}$$

(Equation 2) $$d\left[I\right]/dt=\pi_{I}\left[H\right]\left[V\right]/\left(1+\beta_{I}\left[Ig\right]\left[V\right]\right)-\delta_{I}\left[I\right]-k_{I}\left[I\right]\left[CTL_{R}\right]\text{,}$$

(Equation 3) $$d\left[DC\right]/dt=\lambda_{DC}-\delta_{DC}\left[DC\right]-\pi_{APC}\left(1+\alpha_{recruit}\left[INF1\right]\right)\left(1+\alpha_{APC}\left[Ig\right]\right)\left[DC\right]\left[V\right]\text{,}$$

(Equation 4) $$d\left[APC_{R}\right]/dt=\pi_{APC}\left(1+\alpha_{\text{recruit}}\left[INF1\right]\right)\left(1+\alpha_{APC}\left[Ig\right]\right)\left[DC\right]\left[V\right]-\delta_{APC_{R}}\left[APC_{R}\right]-\mu_{APC}\left[APC_{R}\right]\text{,}$$

(Equation 5) $$d\left[V\right]/dt=\pi_{V}\left[I\right]/\left(1+\beta_{V}\left[INF1\right]\right)-\delta_{V}\left[V\right]-\pi_{I}\left[H\right]\left[V\right]/\left(1+\beta_{I}\left[Ig\right]\left[V\right]\right)-\pi_{APC}\left(1+\alpha_{\text{recruit}}\left[INF1\right]\right)\left(1+\alpha_{APC}\left[Ig\right]\right)\left[DC\right]\left[V\right]-\gamma_{Ig}\left[Ig\right]\left[V\right]\text{,}$$

(Equation 6) $$d\left[IFN1\right]/dt=\sigma_{I}\left[I\right]+\sigma_{APC}\left[APC_{R}\right]-\delta_{IFN1}\left[IFN1\right]\text{.}$$

(Equation 7) $$d\left[CTL_{R}\right]/dt=\mu_{CTL}\left(1+\alpha_{\text{recruit}}\left[INF1\right]\right)\left[CTL_{L}\right]-\delta_{CTL}\left[CTL_{R}\right]\text{.}$$

##### Differentiation of naive CD8^+^ T cells into CTLs in lymph nodes

(Equation 8) $$d\left[APC_{L}\right]/dt=\mu_{APC}\left[APC_{R}\right]-\delta_{APC_{L}}\left[APC_{L}\right]\text{,}$$

(Equation 9) $$d\left[CD4^{+}T_{o}\right]/dt=\lambda_{CD4}-\delta_{CD4}\left[CD4^{+}T_{o}\right]-\pi_{Th1}\left(1+\alpha_{Th1}\left[INF1\right]\right)\left[APC_{L}\right]\left[CD4^{+}T_{o}\right]-\pi_{Tfh}\left(1+\alpha_{Tfh}\left[INF1\right]\right)\left[APC_{L}\right]\left[CD4^{+}T_{o}\right]\text{,}$$

(Equation 10) $$d\left[Th1\right]/dt=\pi_{Th1}\left(1+\alpha_{Th1}\left[INF1\right]\right)\left[APC_{L}\right]\left[CD4^{+}T_{o}\right]-\delta_{Th1}\left[Th1\right]\text{,}$$

(Equation 11) $$d\left[CD8^{+}T_{o}\right]/dt=\lambda_{CD8}-\delta_{CD8}\left[CD8^{+}T_{o}\right]-\pi_{CTL}\left[APC_{L}\right]\left[Th1\right]\left[CD8^{+}T_{o}\right]\text{,}$$

(Equation 12) $$d\left[CTL_{L}\right]/dt=\pi_{CTL}\left[APC_{L}\right]\left[Th1\right]\left[CD8^{+}T_{o}\right]-\delta_{CTL}\left[CTL_{L}\right]-\mu_{CTL}\left(1+\alpha_{\text{recruit}}\left[INF1\right]\right)\left[CTL_{L}\right]\text{,}$$

##### Ig production by pB in lymph nodes

(Equation 13) $$d\left[Tfh\right]/dt=\pi_{Tfh}\left(1+\alpha_{Tfh}\left[INF1\right]\right)\left[APC_{L}\right]\left[CD4^{+}T_{o}\right]-\delta_{Tfh}\left[Tfh\right]\text{,}$$

(Equation 14) $$d\left[B_{o}\right]/dt=\lambda_{B}-\delta_{B}\left[B_{o}\right]-\pi_{pB}\left[APC_{L}\right]\left[Tfh\right]\left[B_{o}\right]\text{,}$$

(Equation 15) $$d\left[pB\right]/dt=\pi_{pB}\left[APC_{L}\right]\left[Tfh\right]\left[B_{o}\right]-\delta_{pB}\left[pB\right]\text{,}$$

(Equation 16) $$d\left[Ig\right]/dt=\pi_{Ig}\left[pB\right]-\delta_{Ig}\left[Ig\right]-\xi_{Ig}\left[Ig\right]\left[V\right]\text{,}$$

### Method details

#### Simulations

The ODEs (Equations 1, 2, 3, 4, 5, 6, 7, 8, 9, 10, 11, 12, 13, 14, 15 and 16) used in the mathematical model of the immune response to SARS-CoV-2 were solved with the LSODA solver in the COPASI biochemical system simulator (v. 4.28) (Bergmann et al., 2017) to obtain the variable and flux time courses. The timestep that was needed to solve the ODEs was automatically chosen by the integrator in the LSODA solver. The concentrations and model parameters used in the simulations are summarized in the following tables. Baseline model parameters listed in Tables 1 and 2 without references were manually adjusted such that the baseline model simulation reproduced the time courses for the clinically observed viral load (Figure 2A) and [Ig] (Figure 2B). Here, a literature value was employed as the initial guessed parameter if it was available from existing literature. Consequently, the baseline simulation was consistent with the clinical data for the DC level 7 months after infection (Pérez-Gómez et al., 2021) (Figure 3). Determination of steady-state solution, linear stability analysis, and sensitivity analysis were also performed with COPASI (Bergmann et al., 2017).

## Data Availability

Data reported in this paper will be shared by the lead contact upon request. The COPASI input data used to generate the data for the current study are available from the corresponding author (TS) upon reasonable request. This paper does not report original code. Any additional information required to reanalyze the data reported in this paper is available from the lead contact upon request.
