# Supplementary material for: Immune response to SARS-CoV-2 in severe disease and long COVID-19
Source: iScience. 2022 Jul 4;25(8):104723. doi: 10.1016/j.isci.2022.104723 (PMC9251893; doi:10.1016/j.isci.2022.104723)
Supplement: Document S1. Figures S1, S2, and Tables S1–S4 [file mmc1.pdf]

iScience, Volume *25*

Supplemental information

Immune response to SARS-CoV-2 in severe disease and long COVID-19

Tomonari Sumi and Kouji Harada

Supplemental Information for

Immune response to SARS-CoV-2 in severe disease and long COVID-19

Tomonari Sumi[1,2] **and Kouji Harada**[3,4]

[1] Research Institute for Interdisciplinary Science, Okayama University, 3-1-1 Tsushima-Naka, Kita-ku, Okayama 700-8530, Japan

[2] Department of Chemistry, Faculty of Science, Okayama University, 3-1-1 Tsushima-Naka, Kita-ku, Okayama 700-8530, Japan

[3] Department of Computer Science and Engineering, Toyohashi University of Technology, Tempaku-cho, Toyohashi 441-8580, Japan

[4] Center for IT-Based Education, Toyohashi University of Technology, Tempaku-cho, Toyohashi, Aichi, 441-8580, Japan

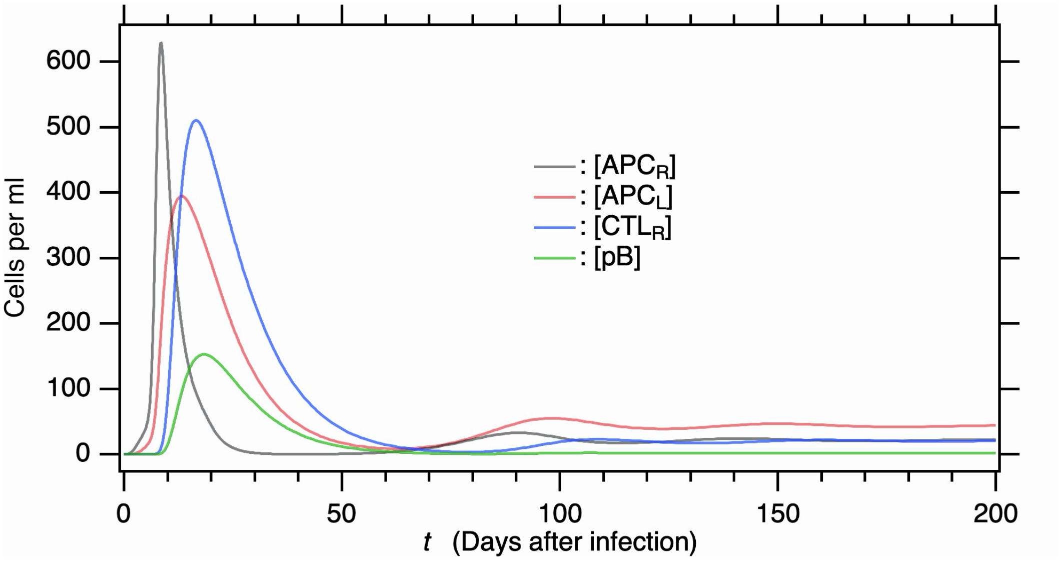

Figure S1. Long-term behavior of immune cell concentrations displayed in Figure 2d.

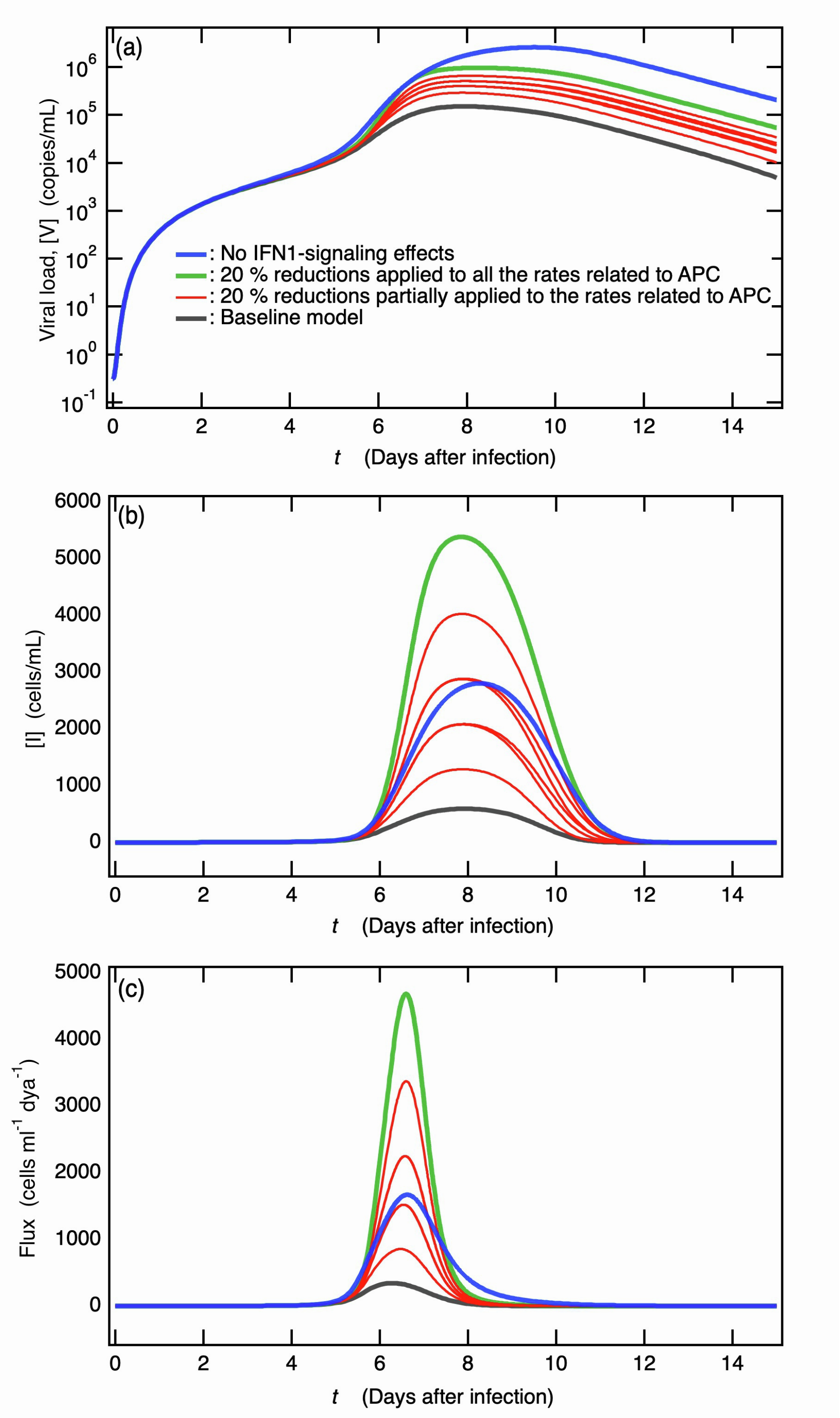

Figure S2. Twenty percent reductions in each rate related to the APC functions result in a large increase in viral infection flux overall, related to information for Figure 4. Time courses of (a) viral load [V], (b) infected cells [I], and (c) the flux of viral infection. In all the figures, the baseline model, 80% APC activity (Model 2), no IFN1 signaling (Model 4), and seven models where 20% reductions in each rate related to APC activities are additively considered from the top to bottom of **STAR★Methods** Table 4, Model 2, are shown. In (b) and (c), the [I] and viral infection flux for the last two of APC activities reduced models (shown as red curves) become higher than the model without IFN1-signaling effects (blue curve), when 20% reductions in each rate related to APC activities are additively taken into considerations one-by-one.

Linear stability analysis based on the eigenvalues of the Jacobian matrix

Table S1. The steady state for the baseline model (STAR★Methods Table 1) with zero value of [V], related to information for Figures 2 and 5. The fixed point for [V] = 0 is unstable in the baseline model.

Type of eigenvalue	Number
Purely real	16
Purely imaginary	0
Complex	0
Zero value	0
Positive real part	1
Negative real part	15

Table S2. Parameter dependent steady state stability for [V] = 0, related to information for Figures 2 and 5. The viral infection rate π_I and viral production rate π_V are reduced from the baseline model (STAR★Methods Table 1, $\boldsymbol{\pi_I}$ = 2.0 × 10^{-6} day^{-1} ml $copies^{-1}$, $\boldsymbol{\pi_V}$ = 700 day^{-1} copies $cells^{-1}$).

Varying viral infection rate $\boldsymbol{\pi_I}$ (day^{-1} ml $copies^{-1}$)	Varying production rate of virus $\boldsymbol{\pi_V}$ (day^{-1} copies $cells^{-1}$)	Stability of the steady state for [V] = 0
2.0 × 10^{-6} × 0.1	700 × 0.1	Unstable
2.0 × 10^{-6} × 0.01	700 × 0.01	Unstable
2.0 × 10^{-6} × 0.001	700 × 0.001	Stable

Table S3. The steady state for the baseline model with a finite value of [V], related to information for Figures 2 and 5. The steady state for the finite value of [V] is asymptotically stable.

Type of eigenvalue	Number
Purely real	10
Purely imaginary	0
Complex	6
Zero value	0
Positive real part	0
Negative real part	16

Sensitivity analysis for the baseline model

Table S4. Sensitivity analysis for the steady state [I] and [V] in the baseline model, related to information for Figure 5.

Model Parameters	Scaled sensitivity to [I]	Scaled sensitivity to [V]
(H production).v	0.245316	0.146659
(DC production).v	-1.83462	-2.83242
(H death).k1	-0.245313	-0.146656
(DC death).k1	0.641779	0.990827
(Virus infection).pi_I	0.245315	0.146658
(Virus infection).beta_I	-0.245105	-0.146531
(APC_R production).pi_APC	-0.641772	-0.990816
(APC_R production).alpha_recruit	-0.151991	-0.234655
(APC_R production).alpha_apc	-0.638908	-0.986394
(Virus production).pi_V	-0.755115	0.147711
(Virus production).beta_V	0.178834	-0.0349818
(Virus death).k1	0.507642	-0.0992994
(IFN1 production by I).sigma	-0.0001767	-0.0009314
(IFN1 production by APC_R).sigma	-0.0775899	-0.408968
(I death).k1	-0.0056177	-0.0033696
(APC_R death).k1	0.673624	0.932026
(APC_R migration).k1	-0.596024	-0.523046
(APC_L death).k1	1.94329	2.38712
(CD4+T0 death).k1	0.908674	1.11621
(Th1 production).pi_th1	-0.199843	-0.112213
(Th1 production).alpha_th1	-0.0060149	-0.0033775
(Th1 death).k1	0.212027	0.127181
(CD4+T0 production).v	-1.03459	-1.27088
(CD8+T0 production).v	-0.239081	-0.143407
(CD8+T0 death).k1	0.212027	0.127181
(CTL_L production).pi_CTL	-0.212025	-0.127179
(CTL_L death).k1	0.0142959	0.00857513
(CTL_L migration).mu_CTL	-0.0142958	-0.008575
(CTL_L migration).alpha_recruit	-0.0033857	-0.0020308
(CTL_R death).k1	0.239083	0.14341
(Kill infedted cells).k_I	-0.239081	-0.143407
(Tfh production).pi_tfh	-0.70882	-1.00398
(Tfh production).alpha_tfh	-0.095209	-0.134855
(Tfh death).k1	0.822575	1.14372
(B0 production).v	-0.82335	-1.14479
(B0 death).k1	0.822575	1.14372

(pB production).pi_pb	-0.822566	-1.1437
(pB death).k1	0.823359	1.14481
(Ig production).pi_ig	-0.82335	-1.14479
(Ig degradation).k1	0.750093	1.04294
(IFN1 degradation).k1	0.0777663	0.409898
(Ig-binding to virus).ksi_ig	0.0732655	0.101869
(Virus neutralized by Ig).gamma_ig	0.0606631	-0.0118664
